# Unlocking play willingness: the dual pathways of curiosity drive and downward social comparison in game advertising

**DOI:** 10.3389/fpsyg.2024.1374649

**Published:** 2024-08-23

**Authors:** Dai Zetian, Xue Juan, Du Jiaxin, Wee Hoe Tan

**Affiliations:** ^1^School of Art, Suzhou University of Science and Technology, Suzhou, China; ^2^Faculty of Social Sciences and Liberal Arts, UCSI University, Kuala Lumpur, Malaysia; ^3^International Institute of Science Diplomacy and Sustainability, UCSI University, Kuala Lumpur, Malaysia

**Keywords:** game advertising, curiosity driven, play willingness, need for cognitive closure, downward social comparison

## Abstract

The integrity and clarity of information have long been regarded as the cornerstones of advertising strategy. However, recent game advertising has taken a different approach. Specifically, incomplete game videos, especially those showcasing losing gameplay, are more likely to stimulate players’ interest compared to complete videos of winning gameplay. This study, through five experiments, uncovers a dual-pathway mechanism behind this phenomenon. Firstly, information gaps resulting from incomplete videos trigger curiosity drive, compelling viewers to seek more information and reinforcing their willingness to engage in gaming. Secondly, witnessing failures in game demonstrations activates components of downward social comparison and competitive motivation. These findings offer valuable insights into the complex dynamics of game advertising, shedding light on the effects of information gaps, curiosity, and social comparison. They provide valuable implications for advertising strategies within the gaming industry.

## 1 Introduction

Video games are used in a variety of fields, which include computer science (Wong et al., 2014), pedagogical aids (Tan et al., 2010; Adnan et al., 2019), and cultural communication (Chen, 2018). Among these usages, media publicity is an important vehicle for promoting the use of game. Advertising targets on people based on their web-browsing habits as mobile internet usage increases (McStay, 2017; Gong et al., 2021). The advertisements showcase as many in-game highlights as they can in order to improve download rate (Seo et al., 2018). Normally, the key strategy of game advertising is to provide complete and understandable information. However, recent advertisements that involve mobile games appear not to show the entire message purposely and hide the in-game narrative outcome, such as obtaining necessary equipment and eliminating the boss.

On the other hand, in addition to the purposive lack of information, game advertisements seem to prefer using negative gaming experiences to capture players’ attention. In some cases, even within simple games, players were intentionally led to execute actions leading to failure, rendering it as an impossible mission to complete the game successfully. This phenomenon appears to be inconsistent with the strategies commonly employed in advertising design, which typically involve showcasing a product’s positive aspects (Kim and Kim, 2021). Similarly, based on research, witnessing others’ success has a positive impact on individuals (see Eccles and Wigfield, 2020; Schunk and DiBenedetto, 2020), and successful behavior tends to induce consumer imitation (Lee and Kim, 2022).

Building upon the analysis mentioned above, this study aimed to uncover the mechanism and rationale behind the use of information gaps and negative gaming experiences in game advertisements. This study seek to address the following questions: Why does the absence of information relative to complete information tend to stimulate players to download the game? And does observing others’ gaming failures enhance the viewer’s interest in gaming? In this context, this study was grounded in the concept of cognitive closure needs, while also considering downward social comparison as both a moderating and mediating variable, with the goal of revealing the psychological mechanism underlying this phenomenon.

### 1.1 Curiosity and advertising

The term “curiosity” refers to an innate desire to learn and it is considered to be a relatively stable cognitive tendency (Kashdan and Silvia, 2009). Perceptual and epistemic curiosity are divided into two categories by Berlyne (1966). Epistemic curiosity is the need or desire to learn something in order to dispel uncertainty (Berlyne, 1966; Litman and Spielberger, 2003; Collins et al., 2004; Litman, 2005; Kang et al., 2009).

Generating epistemic curiosity in consumers is gradually becoming one of the strategies used in advertising. Advertisements that stimulate curiosity typically contain one or more particularly appealing elements, referred to as curiosity-evoking elements (Hornikx and Mulder, 2015). When advertising campaigns stimulate consumers to experience curiosity, further emotional and cognitive processes are initiated (Hill et al., 2016). The use of incomplete information and ambiguity in promotion makes the message lack a clear meaning or outcome, which leads consumers to complement the message through purchase actions (Daume and Hüttl-Maack, 2020). In this process, allowing curiosity raises the individual’s positive expectations of the product, would be resulting in a positively biased evaluation (Toteva et al., 2021).

Similarly, games capitalise on players’ curiosity to increase their willingness to play. Nevertheless, unlike the commodity, games are a type of entertainment experience (Oliver et al., 2016), giving players a dynamic and unknown environment to explore (Galdieri et al., 2020). Thus, curiosity in games is primarily driven, which includes the control of information flow (backstory) (Atkins, 2003; Wouters et al., 2011), as well as game rewards (Nagle et al., 2014; To et al., 2016). However, little research has been conducted to explore the impact of watching others play on curiosity and the role that curiosity plays in promotional videos for games. Considering this, the researcher proposes the following hypotheses.

*H1:* Game videos with missing information are more likely to stimulate play willingness than game videos with complete information.

*H2:* Curiosity plays a mediating variable in the effect of missing information on play willingness.

### 1.2 Need for cognitive closure

Need for Cognitive Closure (NFCC) refers to the desire and motivation to find a clear answer to a question, regardless of what the answer involves, that has a precise meaning and can be categorised (Webster and Kruglanski, 1994). NFCC can influence an individual’s social judgment and interpersonal phenomena (Pierro and Kruglanski, 2008). Webster (1993) examined the relationship between cognitive closure and over-attribution bias—defined as the tendency to attribute too much causality or significance to a single factor while overlooking other contributing factors—and found that this bias was higher in high cognitive closers than in controls and low cognitive closers. Webster and Kruglanski (1994) showed that high cognitive closers are more likely to fall prey to the ‘primacy effect’.

According to Kruglanski (2013) and Kruglanski et al. (1993), individuals with high NFCC prefer heuristic, simplistic and top-down information processing and therefore prefer expectancy-consistent information during hypothesis generation and testing. This allows individuals to quickly accept the first hypothesis for closure and ignore subsequent inconsistent information. However, other studies have demonstrated that high NFCC individuals prefer to process expectancy-consistent information to reduce uncertainty (Kemmelmeier, 2015).

Based on the above analysis, game videos with incomplete information may activate players’ need for cognitive closure due to the ambiguous and uncertain outcomes. According to Kruglanski (2013), individuals with high NFCC are more intolerant of ambiguity and lack information than individuals with low NFCC. Hence the following hypothesis:

*H3:* NFCC plays a moderating role in the information gap affecting player curiosity. Specifically, the high NFCC group with the elevation of curiosity-driven by information deficit is significantly stronger than the low NFCC group.

### 1.3 Success and failure in games

In the realm of game design, encouraging players to pursue success while avoiding failure has long been regarded as an essential aspect (Connolly et al., 2012). Employing various tactics to convey these concepts, different game genres often use game outcomes, goal achievement, and task completion as common indicators of success and failure within the gaming context (Apperley, 2006). Achieving victory in a game typically results in an elevated emotional state for players, leaving them feeling more satisfied and exhilarated than when they began.

Conversely, facing unfavorable game results can evoke feelings of frustration among players, and in certain instances, it may even lead players to prematurely discontinue their gaming experience (Steffen et al., 2013). This is predominantly attributed to the lack of timely positive feedback, which diminishes players’ engagement with the game (Paiva et al., 2020). However, it is worth noting that the discourse on success and failure, primarily centered around the player’s perspective, often fails to consider the influence of witnessing the successes and setbacks of others on the behavior and motivation of observers.

The psychological impact of observing others’ failures is significantly evident in consumer behavior. Moisieiev et al. (2020) demonstrated that witnessing the failures of competing brands enhances consumers’ satisfaction with their own choices. This phenomenon is particularly pronounced in advertising, as studies by Yucel-Aybat and Kramer (2017, 2018) revealed that showcasing the shortcomings of competitors can positively influence attitudes and purchase intentions towards the advertised brand (Moisieiev et al., 2020). This reflects a psychological “contrast effect,” where comparisons amplify positive evaluations of one’s choices.

Conversely, in the field of educational psychology, observing the success of others plays a crucial role. As Bandura (1982) and Schunk and DiBenedetto (2020) noted, students acquire knowledge and skills through social learning by observing others’ successes, thereby enhancing their self-efficacy.

However, the applicability of these psychological theories varies in the context of gaming. As Oliver et al. (2016) identified, gaming is primarily an entertainment experience, not a compulsory task. Research by Eccles and Wigfield (2020) and Sankey and Machin (2014) suggest that even with confidence in task completion, individuals might lack sufficient motivation to engage in non-compulsory tasks. This implies that in gaming, confidence in winning does not necessarily translate into motivation for personal trial, especially when the game objectives diverge from the goal-oriented efforts seen in educational settings.

In gaming, players’ core motivations often align with fulfilling basic psychological needs, such as demonstrating competence, forming social connections, and achieving competitive victories (Przybylski et al., 2010; Xi and Hamari, 2019). Therefore, witnessing others’ success in games, particularly in simpler ones, might diminish the necessity to prove one’s abilities through personal attempts.

Therefore, the following hypothesis was proposed:

*H4:* Compared to successful game demos, unsuccessful ones are more effective at reinforcing an individual's play willingness.

### 1.4 Downward social comparison and competitive motivation

Social comparison, defined as the process of evaluating one’s own abilities, attributes, or situations in relation to others, is categorized into three distinct types: upward, downward, and horizontal. Horizontal social comparison, originally identified by Festinger (1954), occurs between individuals who perceive themselves to be at a similar level. This type of social comparison promotes realistic self-evaluations and can enhance feelings of camaraderie and belonging, as it often involves comparisons with peers who share similar status or conditions. These comparisons are not only based on current situations but also on anticipated future status, providing a comprehensive framework for understanding one’s place within a social or professional hierarchy (Lee and Griffith, 2019; Pahlevan Sharif et al., 2022). Upward social comparison involves comparing oneself to those perceived as better off, often motivating self-improvement but potentially lowering self-esteem. Downward social comparison involves comparing oneself to those perceived as worse off, often boosting self-esteem and fostering competitive behavior, especially after observing others’ failures (Diel and Hofmann, 2019; Diel et al., 2021).

However, the different attribution styles of observers towards others’ success and failure form the basis for social comparison (Weary et al., 1987; Salanova et al., 2012). Individuals typically attribute their own successes to internal factors, such as their abilities or effort, and their failures to external circumstances beyond their control. Conversely, observers tend to attribute the successes of others to external factors, such as luck, and their failures to internal factors, such as a lack of ability or effort. This difference in attribution leads observers to make downward comparisons after witnessing others’ failures, strengthening their self-evaluation (Hauke and Abele, 2020). Therefore, game advertisers may take advantage of this difference in attribution, preferring to show videos of novice gameplay to reinforce players’ downward social comparison and competitive motivation, thereby increasing their willingness to play.

Herewith two specific hypotheses:

*H5:* The impact of the game outcome in the advertisement on the willingness to download is mediated by Downward Social Comparison (DSC).

*H6:* The impact of the game outcome in the advertisement on the willingness to download is mediated by Competitive Motivation (CM).

### 1.5 Research overview

This research series systematically investigates the psychological mechanisms by which game advertising influences play willingness, driven by curiosity and downward social comparisons. Study 1 establishes the foundational effect by demonstrating how the omission of information in game videos enhances players’ willingness to engage through the activation of their curiosity drive. Building on this, Study 2 confirms that the absence of clear game outcomes further amplifies players’ willingness to play, using a mediating model to illustrate how curiosity bridges the gap between information omission and increased player willingness. Study 3 introduces the NFCC as a moderating factor, examining how individual differences in NFCC influence responses to the incomplete information presented in game advertisements. Study 4 then explores how specific game outcomes can drive player willingness through mechanisms of downward social comparison and competitive motivation, emphasizing the role of perceived successes and failures. Finally, Study 5 synthesizes the insights from previous studies by analyzing a dual-path mechanism where both curiosity and downward social comparison independently contribute to enhancing the play willingness.

## 2 Study 1

The objective of Study 1 was to verify that the information missing from the game video had an effect on the play willingness. Therefore, a hypothesis is proposed: Compared with the complete video, the video that does not show the ending is more likely to stimulate play willingness.

According to G*Power (Faul et al., 2007), for moderate effect size (ANOVA, *f* = 0.2), 246 subjects were required to achieve a statistical validity of 1−*β* = 0.8. The study planned to recruit 25% more subjects than the calculated sample size for each online experiment, thus yielding a reasonable subject size of at least 300 for study1.

### 2.1 Experimental design

**Figure 1:**
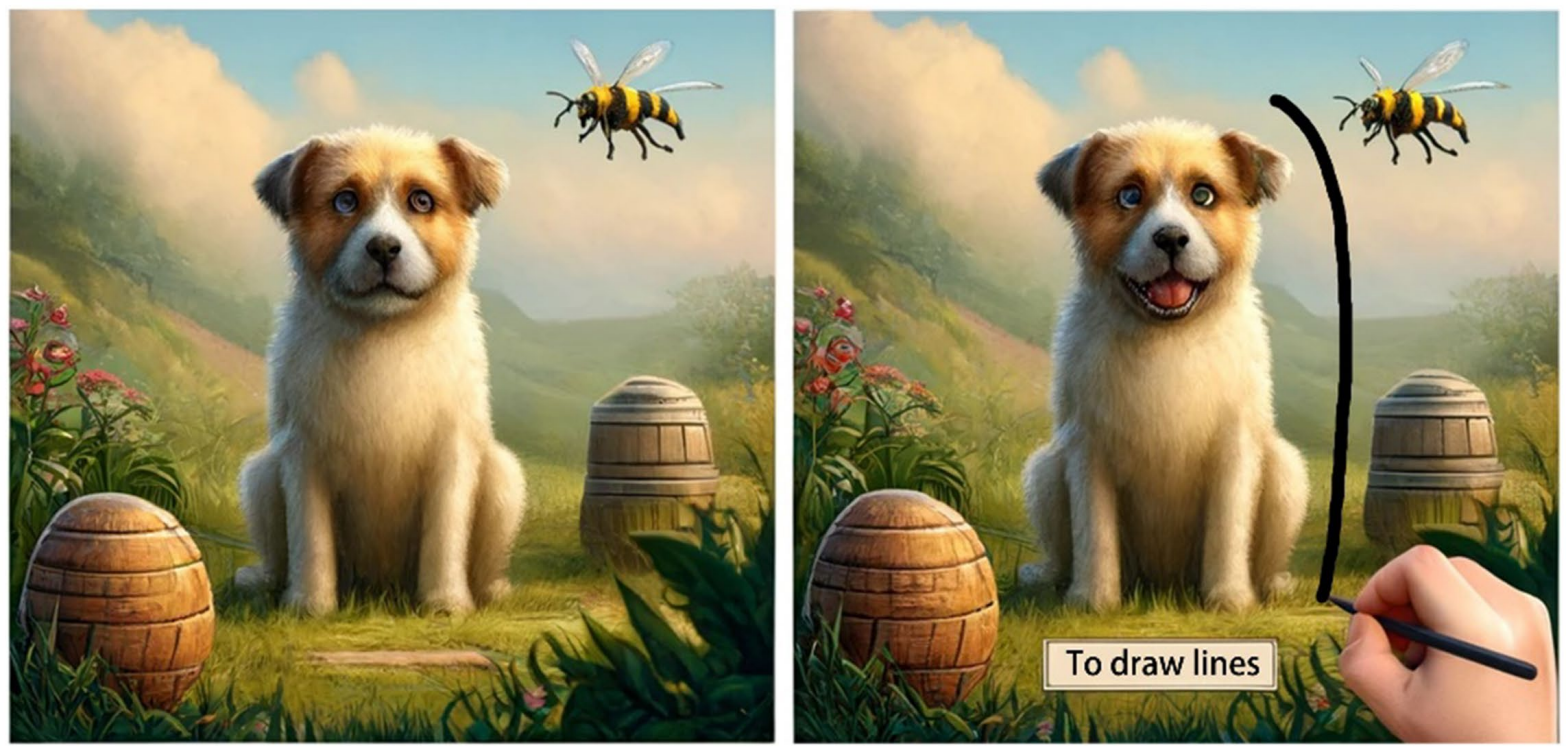
Game videos of Study 1. Note: Images generated by ChatGPT, version 4.0, response to “(Please create two illustrations of the game to protect your puppy from wasps. 1. The wasp flies to the dog. 2. The wasp is blocked by lines drawn by the player, protecting the dog from harm and displaying ‘To Draw Lines’)”, OpenAI (22 May 2024).

300 undergraduate students from an anonymous university were recruited and paid for the experiment (144 females, 156 males; age, M = 22.7, SD = 3.1). The respondents were randomly divided into three groups (Display/Undisplay/Demo), and each watched a 10-s game video (see Figure 1), which shows a mobile game in which the player has to draw lines to protect the puppy from the bees (detail in Figure 1).

The respondents were unaware whether the drawing method shielded the puppy from the bees because the results were not visible in the undisplayed group’s video. The video for the display group, however, is completed. The demo group’s video only demonstrates the rules of the game. The videos from all three groups are on the same level, each lasting 10 s.

As Table 1 shows that, after watching the video, the respondents were asked to rate their play willingness for the game, i.e., “I want to try this game” (0 = “totally disagree,” 10 = “Completely agree”). Furthermore, the participants responded to question 3, “Have you ever played this type of game?” in order to prevent the impact of gaming experience on the experiment’s outcomes. (1 = played, 2 = did not play). In addition, question 2 was set to exclude invalid samples in order to determine the accuracy of the subjects’ perceptions of the outcome of the game, i.e., “Did the puppy avoid bee attacks due to drawing lines? (1 rescued /2 not rescued/3 no result, only demonstration of play).” Finally, subjects were asked to complete measures of demographic variables (Q3 & Q4).

**Table 1:** Questionnaire for play willingness in Study 1.

Data collection	Question	Answers
Play willingness	1. I want to play this game (0–10; 0 = “Strongly disagree,” 10 = “Strongly agree”)	
Perception of Game result	2. Did the puppy avoid bee attacks due to drawing lines? (1 = Yes /2 = No / 3 do not know, only gameplay demo)	
Game experience	3. Have you ever played this game? (1 = Yes / 2 = No)	
Demographic variable	4. What is your gender (1 = Male / 2 = Female)?	
	5. How old are you?	

### 2.2 Analysis methods

Linear regression analysis was used to determine the correlation between the statistical variables (age and gender) and the play willingness. When the results were significantly correlated, the statistical variables were brought into the multi-factor ANOVA as covariates. When there was no correlation, ANOVA tests were carried out directly with game outcome (success/failure/control) as the independent variable and willingness to download as the dependent variable.

### 2.3 Result of Study 1

The results showed that all participants could accurately identify the game result (question 2). Nevertheless, 16 respondents were disqualified because they had played this game. In light of this, the game result, gender and age, were used as independent variables in a regression analysis, while the game-playing willingness was used as the dependent variable. The results showed that the effect of gender differences on willingness to play was not significant, Male: *M* = 5.975, *SD* = 1.126; Female: *M* = 5.912, *SD* = 1.116; *F*(2, 300) = 0.219, *p* = 0.640. There is also no significant difference in the willingness to play between respondents of different ages under the same conditions, *F*(15, 300) = 0.517, *p* = 0.930. And there was also no interaction between gender and age, *F*(11, 300) *=* 0.973, *p =* 0.480. Therefore, demographic variables (age and gender) were not used as predictor variables and an ANOVA was conducted with game control (Display/Undisplay/Demo) as the independent variable and play willingness as the dependent variable.

As Table 2 shows that, game result has a significant effect on play willingness, *F*(1, 300) = 48.993, *p* < 0.001, *ηp^2^* = 0.247. The play willingness in the undisplay group to was significantly higher than the display group, Undisplay: *M* = 6.750, *SD* = 1.115; Display: *M* = 5.170, *SD* = 1.115, *MD* = 1.580, *p* < 0.001. Moreover, the group of undisplay’s willingness to play is significantly higher than the group of demos, Demo: *M* = 5.723, *SD* = 1.114, *MD* = 1.027, *p* < 0.001. Finally, the group of demos is significantly higher than the group of display, *MD* = 0.553, *p* < 0.001.

**Table 2:** Multiple comparisons in Study 1.

Control (I)	Control (J)	MD (I-J)	SE	*P*	Lower bound	Upper bound
Demo	Display	0.553	0.161	0.001	0.235	0.871
Undisplay	Display	1.580	0.162	0.000	1.261	1.899
Undisplay	Demo	1.027	0.161	0.000	0.709	1.345

### 2.4 Discussion of Study 1

According to the results of Study 1, videos that do not display game results are more likely to promote people to play. This implies that the lack of information could indeed better stimulate individuals’ play willingness than complete information. Moreover, demonstrating the game process is a better promotional strategy than demos. Therefore, the researcher speculates that full videos allow players to gain more information and thus clarify their perception of the game, which reduces players’ curiosity. This implies that hiding the game’s outcome in promotional videos is a great tactic for attracting players’ interest. Conversely, showing the game’s ending directly would have dampened players’ curiosity to some extent, even less so than just demonstrating the game’s rules (demo).

## 3 Study 2

The aim of Study 2 was to identify the psychological mechanisms through which information missing influences play willingness. Therefore, it is proposed that “curiosity-driven” is a mediator of game results that influences player willingness.

### 3.1 Research design

**Figure 2:**
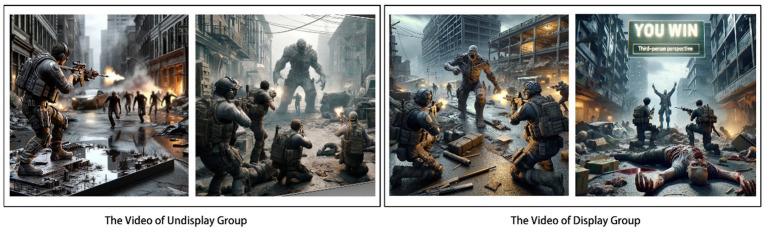
Game videos of Study 2. Note: Images generated by ChatGPT, version 4.0, response to “(Please make four game illustrations: 1. A soldier shoots zombies. 2. Three soldiers shoot zombies. 3. Six soldiers shoots zombie BOSS. 4. Six soldiers killed zombie BOSS, and YOU WIN appears)”, OpenAI (22 May 2024).

In Study 2, a zombie fighting game was used as a study. The player controls a soldier who shoots zombies and gets bonus upgrades to kill bosses by moving around (see Figure 2). Unlike study 1, the video of this game is an actual advertisement. The video was recorded and edited, with the end of the advert cut out for the experimental group and the full version for the control group. A total of 306 students (Age: M = 26.3, SD = 3.2; 164 females, 142 males) were sampled for this experiment after excluding participants who could not identify the results of the game correctly and had experience with the game (*n* = 24).

This experiment was more focused on identifying the psychological mechanisms by which game results affect play willingness. Therefore, the demo group was removed in order to compare information missing and complete information. After watching the game video, each participant was required to complete a questionnaire that provided feedback about play willingness, perception of the game’s outcome, game experience, gaming-related curiosity, and demographic information (details in Table 3). Among these, curiosity driven contained three questions, which included three player curiosity tendencies. As a result, the average of the three question scores was used as the quantified index of curiosity driven because the consistency analysis revealed that all three questions had consistency levels above 0.85.

**Table 3:** Questionnaire for play willingness in Study 2.

Items	Question	Answers
Play willingness	1. I want to play this game (0–10; 0 = “Strongly disagree,” 10 = “Strongly agree”)	
Perception of game result	2. Does the end of the video show the ending of the fight against the boss? (1 = Yes / 2 = No)	
Game experience	3. Have you ever played this game? (1 = Yes / 2 = No)	
Curiosity driven	4. I’m curious about how it actually feels to play this game (0–10; 0 = “Completely Disagree,” 10 = “Completely Agree”)	
	5. I wonder what would happen if I played this game (0–10; 0 = “Completely Disagree,” 10 = “Completely Agree”)	
	6. I’m interested in the game’s level design (0–10; 0 = “totally disagree,” 10 = “totally agree”)	
Demographic variable	7. What is your gender? (1 = Male / 2 = Female)?	
	8. How old are you?	

### 3.2 Data analysis

The PROCESS macro for SPSS was used as the analysis tool for study3. Based on the bootstrapping method proposed by Hayes (2017) and Bolin (2014), Model 4 in Process was selected as the mediating effect hypothesis model. Game Result was used as the independent variable, curiosity-driven as the mediating variable, play willingness as the dependent variable, and gender and age as covariates for mediating effects analysis. The bootstrap sample size was set at 5000, and the confidence interval was set at 95%.

### 3.3 Result of Study 2

A sensitivity power analysis using G*Power indicated that the sample size of 306 can detect a Cohen’s d of 0.321, categorized as a small to medium effect (Allen, 2011). This shows that, even with a small effect size, the sample size of 306 has enough statistical power (80%) to detect such effects in an independent samples *t*-test. As a result, it can be concluded that 306 samples are appropriate for Study 2.

A mediating effects analysis was conducted with Game Result as the independent variable, curiosity-driven as the mediating variable and play willingness as the dependent variable. As Table 4 shows that, throughout the regression equation, gender did not predict play willingness (*β* = 0.160, *p* = 0.722) and had a non-significant effect on curiosity (*β* = −0.003, *p* = 0.951). Similarly, age did not predict play willingness (*β* = 0.005, *p* = 0.908) and curiosity (*β* = 0.270, *p* = 0.635).

**Table 4:** Intermediary effects model testing.

	Willingness (Total)			Willingness			Curiosity-driven		
	*t*	*β*	*P*	*t*	*β*	*P*	*t*	*β*	*P*
Gender	0.356	0.160	0.722	0.291	−0.009	0.771	−0.061	−0.003	0.951
Age	0.116	0.005	0.908	0.331	−0.003	0.741	0.475	0.270	0.635
Game Result	7.618	0.379	0.000	9.594	0.512	0.000	4.612	0.262	0.000
Curiosity Driven	9.700	0.413	0.000						
R^2^	0.395			0.237			0.069		
F	76.614			30.910			7.597		

Overall in the regression equation, the game result had a significant effect on play willingness (*β* = 0.270, *p* < 0.001); Curiosity-driven predicted play willingness significantly (*β* = 0.413, *p* < 0.001). On the other hand, in the equation with play willingness as the independent variable and game result as the predictor variable, the game result had a significant effect (*β* = 0.512, *p* < 0.001). Finally, in the regression equation with Curiosity as the dependent variable, the game result effectively predicted Curiosity (*β* = 0.262, *p* < 0.001).

As Table 4 show that game result has a significant positive effect on play willingness, R^2^ = 0.395, *F*(1, 305) = 76.614, *p* < 0.001. In the whole regression equation, the proportion of indirect effect is 0.22, CI = [0.186, 0.466]. The direct effect ratio was 0.78, CI = [0.813, 1.380]. Therefore, the original hypothesis is confirmed that curiosity-driven plays a mediating role (see Table 5).

**Table 5:** Indirect Effects-Direct Effects-Total Effect for Study 2.

	Effect	Boot SE	LLCI	ULCL	Path	Ratio
Indirect	0.913	0.070	0.186	0.466	Game result—curiosity-driven—play willingness	65%
Direct	0.496	0.144	0.813	1.380	Game result—play willingness	35%
Total	1.409	0.147	1.120	1.698		

### 3.4 Discussion of Study 2

The impact of information missing (Display/Undisplay) on play willingness was once again confirmed by Study 2. Remarkably, the player’s play willingness increases when the game’s outcome is invisible. Study 2 also shows that the effect of ‘game results’ on the willingness to play is partially (and not fully) mediated. When the video does not show the ending, the player’s curiosity is not satisfied. Curiosity, as an intrinsic drive to play, increases the player’s willingness to play in order to satisfy curiosity about the end of the game.

As discussed above, the absence of a game’s outcome may create a perception of ambiguity and uncertainty about the solution, which perception may activate the observer to seek a clear answer; this is called NFCC (Webster and Kruglanski, 1994). Similarly, NFCC can also be used as a personality trait (Roets et al., 2015), which leads to differences in the curiosity drive of each player. Therefore, in the follow-up experiment, this study hypothesized that NFCC, as a moderating variable, might influence the game result-curiosity-driven process. Study 3 was designed to test this hypothesis.

## 4 Study 3

Study 3 was conducted to examine the NFCC moderating effects on the willingness of players to watch video of a game. For this reason, hypothesis 3 was tested: For the low NFCC, the elevation of curiosity-driven by information deficit was much less strong than the difference for the high NFCC group.

### 4.1 Research design

**Figure 3:**
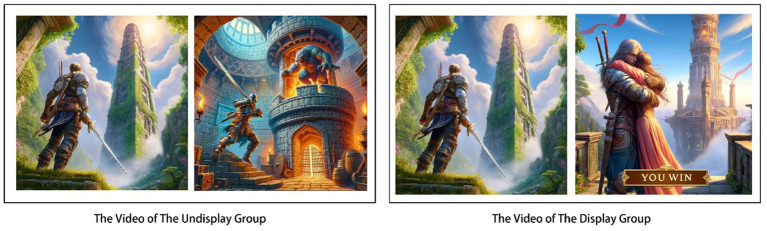
Game videos of Study 3. Note: Images generated by ChatGPT, version 4.0, response to “(Generate three game illustrations. 1. The warrior at the bottom of the tower. 2. The warrior fights the monster in the middle of the tower. 3. The warrior saves the princess, and YOU WIN appears)”, OpenAI (22 May 2024).

300 undergraduate students from XX University were recruited and paid for the experiment (144 females, 156 males; age, M = 22.7, SD = 3.1). They were instructed to watch a video of a tower climbing game. The video showed the soldiers destroying the enemies on each platform of the tower in order to obtain more advanced equipment, finally reaching the top and rescuing the princess. The experimental group watched the video without the ending where the soldier climbs to the top, while the control group was shown the full video (detail in Figure 3). At the end of the 20s video, participants were asked to fill out a questionnaire and later to complete the Need for Closure Scale (NFCS; 1994).

#### 4.1.1 The questionnaire for Study 3

Perceptions of game results and game experience were used in the questionnaire as screening questions to weed out those who did not fit the criteria. Furthermore, as the outcomes of Studies 1 and 2 demonstrated, play willingness, and curiosity-driven behaviour were not significantly influenced by gender or age. Consequently, in Study 3, demographic variables were eliminated (detail in Table 6).

**Table 6:** Questionnaire for play willingness in Study 3.

Data collection	Question	Answers
Play willingness	I want to play this game (0–10; 0 = “Strongly disagree,” 10 = “Strongly agree”)	
Perception of game result	Does the video show the soldier getting the final equipment (1 = Yes /2 = No)	
Game experience	Have you ever played this game? (1 = Yes /2 = No)	
Curiosity driven	I’m curious about how it actually feels to play this game (0–10; 0 = “Completely Disagree,” 10 = “Completely Agree”)	
	I wonder what would happen if I played this game (0–10; 0 = “Completely Disagree,” 10 = “Completely Agree”)	
	I’m interested in the game’s level design (0–10; 0 = “totally disagree,” 10 = “totally agree”)	

#### 4.1.2 The need for cognitive closure scale

At the end of the questionnaire, subjects were measured on the NFCC, administered using The Need for Closure Scale (NFCS) published by Webster and Kruglanski (1994). Because the NFCS has been found to predict a wide range of critical social cognitive processes, its high reliability and validity have been widely recognised and translated into different languages (Pierro et al., 1995; Chiu et al., 2000). It should be noted that to unify scale dimensions and eliminate the impact of non-standardized data, the mean value of all items was used as the study’s final outcomes. Additionally, the subjects’ ratings varied uniformly between 1 and 10. (1 being strongly disagree and 10 being strongly agree).

### 4.2 Result of Study 3

A G*Power sensitivity analysis showed that a sample size of 300 can find a Cohen’s d of 0.324, which is considered a small to medium effect (Allen, 2011). Therefore, the sample size of 300 has enough statistical power (80%) to find effects.

To determine whether the experimental group influenced NFCC, an independent samples *t*-test was conducted. The results show that there is no significant difference in NFCC between the two groups (*t* = 0.117, df = 298, *p* = 0.907), indicating that the game video without an ending did not affect NFCC (see Table 7).

**Table 7:** Main effect test (Game result--play willingness).

Curiosity driven (M)	coeff	se	*t*	*P*	LLCI	ULCI
Constant	4.156	0.202	20.537	0.000	3.757	4.554
Game result	1.0867	0.145	7.481	0.000	0.801	1.373
*R* ^2^	0.159					
*F*	55.967					

As in the previous experiment, the main effect of game results (Display/Undisplay) on play willingness was significant, *F*(1, 299) = 55.967, *p* < 0.001, R^2^ = 0.159. Respondents who watched the video without displaying the result had a higher willingness to play (Display: M = 4.787, SD = 1.380; Undisplay: M = 6.428, SD = 1.400).

**Figure 4:**
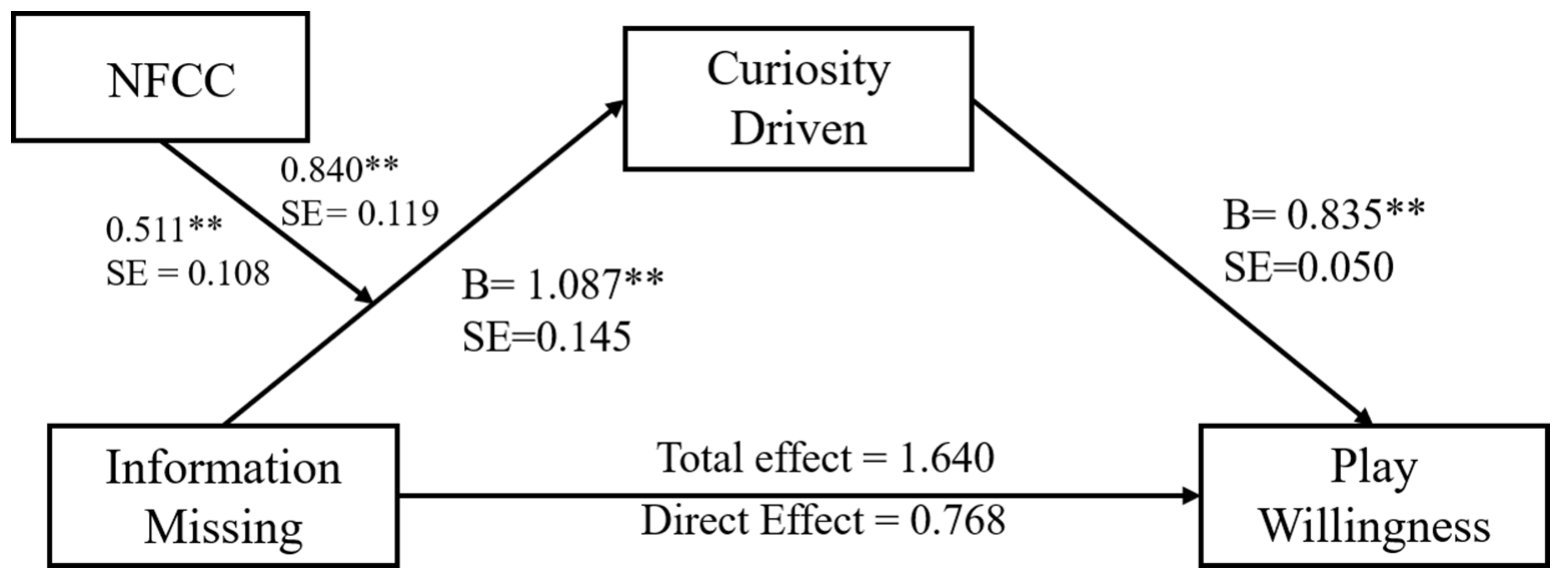
Map of the intermediary tests with moderation.

As Table 8 shows that curiosity driven played a mediating effect in the whole regression equation, with an Indirect effect = 0.872, CI = [0.613, 1.160]. In this process, the influence of game result on curiosity driven was statistically significant, *F*(1, 299) = 8.879, *β* = 0.405, *p* < 0.001; curiosity driven had a significant effect on play willingness, *F* (20, 299) = 16.527, *β* = 0.626, *p* < 0.001 (see Figure 4).

**Table 8:** Indirect Effects-Direct Effects-Total Effect for Study 3.

	Effect	se	*t*	*P*	LLCI	ULCI
Total	1.640	0.180	9.146	0.000	1.287	1.993
Direct	0.768	0.160	4.810	0.000	0.454	1.082
Indirect	0.872	0.140	/	/	0.613	1.160

**Figure 5:**
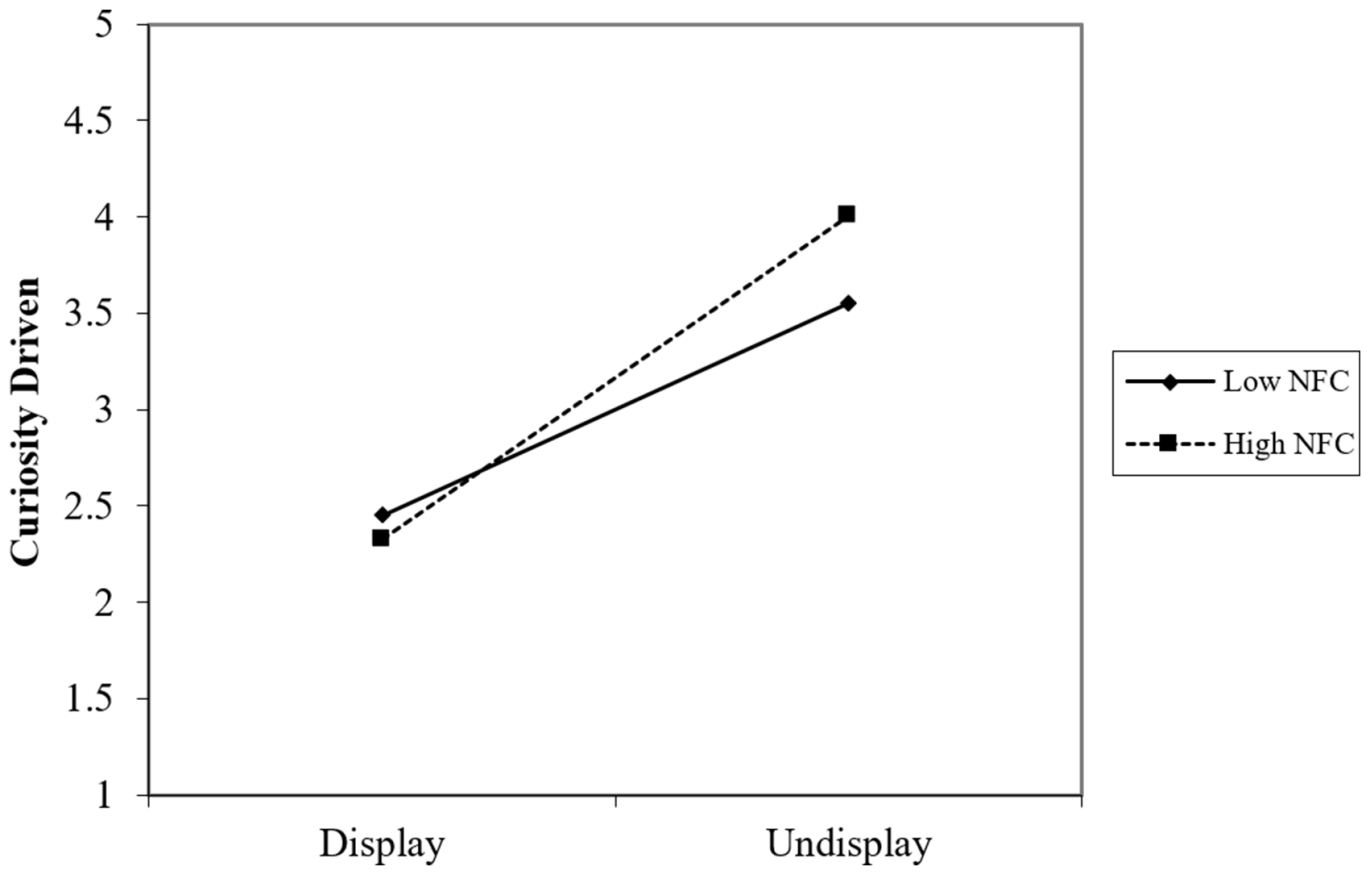
The moderating effect of NFCC is in “Game Result – Curiosity Driven.”

The moderating effect was tested with Game result as the independent variable, NFCC as the moderating variable and Curiosity Driven as the dependent variable. As shown in Table 9, the regression equation is very significant, R^2^ = 0.813, *F*(291, 299) =313.203, *p* < 0.001. The influence of NFCC on Curiosity Driven was statistically significant (*t* = 24.223, SE = 0.025, *p* < 0.001). The influence of Game result on Curiosity driving was also significant *t* = 15.909, SE = 0.069, *p* < 0.001. The interaction between NFCC and Game result was significant (*t* = 5.831, SE = 0.049, *p* < 0.001) (see Figure 5).

**Table 9:** NFCC moderating effect test.

Curiosity driven	coeff	se	*t*	*P*	LLCI	ULCI
Constant	5.788	0.035	166.950	0.000	5.719	5.856
NFCC	0.596	0.025	24.223	0.000	0.548	0.644
Game result	1.103	0.069	15.909	0.000	0.967	1.239
int_1	0.287	0.049	5.831	0.000	0.190	0.384
*R* ^2^	0.813					
*F*	313.203					

As shown in Table 10, the mediated model with moderating variables was highly significant, Effect size = 0.230, SE = 0.039, CI = [0.155, 0.310]. In respondents with higher NFCC (M + SD =1.800), game result had a significant positive predictive effect on curiosity-driven, simple slope = 0.840, SE = 0.119, *p* < 0.001; in respondents with lower NFCC (M-SD = −1.800), the game result had the same statistically significant predictive effect on curiosity-driven, simple slope = 0.511, SE = 0.108, *p* < 0.01. The positive effect of videos that did not show the game result on play willingness increased with the enhancement of individual NFCC. In contrast, as NFCC decreased, the difference caused by Display and Undisplay on play willingness gradually decreased.

**Table 10:** Intermediary tests with moderation.

NFCC	Effect	Boot SE	LLCL	ULCL
−1.800	0.471	0.108	0.237	0.698
0.000	0.885	0.088	0.721	1.066
1.800	1.300	0.119	1.069	1.534
Moderated mediation	0.230	0.039	0.155	0.310

### 4.3 Discussion of Study 3

Study 3 reconfirmed the main effect of information missing on play willingness. Moreover, similar to study 2, the positive effect of Curiosity Driven as a mediating variable on play willingness was highly significant. Notably, the individual NFCC was able to moderate the effect of information missing on curiosity driven, and thus the slope of the indirect effect. Specifically, for the low NFCC in review group, the elevation of curiosity-driven by information deficit was much less strong than the difference for the high NFCC group. Therefore, it is more important for individuals with HIGH NFCC to avoid falling into the propaganda trap brought about by the lack of information in the game video.

## 5 Study 4

Study 4 aimed to further examine the influence of game outcomes (success/failure) on play willingness and whether downward social comparison and competitive motivation act as mediating variables. In this regard, the study hypothesized that a failed game outcome would have a more significant positive impact on play willingness compared to a successful game outcome. In this process, game outcomes were believed to affect Downward Social Comparison (DSC), subsequently influencing Competitive Motivation (CM).

### 5.1 Research design

The zombie game advertisement video was edited for use in Study 2. Unlike Study 2, both the experimental group and the control group retained the game outcome. However, in the experimental group, the demonstration player was ultimately defeated by the boss, while in the control group, the demonstration player defeated the final boss. Additionally, during the gameplay, the demonstration player in the experimental group deliberately performed low-quality actions and did not select powerful buffs and weapons, resulting in an inability to defeat the final boss. In contrast, the demonstration player in the control group did the opposite and successfully defeated the final boss.

In Study 4, a total of 360 participants contributed valid data (Age: M = 28.7. SD = 5.3; 164 females, 142 males). At the end of the 20-s advertisement, participants were asked to complete a Likert survey, which included play willingness, downward social comparison, and competitive motivation. Regarding Downward Social Comparison (DSC), references were made to the studies by Brown et al. (2007) and Gibbons et al. (2002). For example, questions like “Compared to the players in the video, I think I can…” and “Compared to the players in the video, I think my gaming skills are…” were measured using a 7-point Likert scale (1 = “far worse,” 7 = “much better”). The measurement of competitive motivation was adapted from the research by Tjosvold et al. (2006) and Cagiltay et al. (2015). For instance, questions such as “I feel very confident to play better,” “I want to do my best to outperform the players in the video,” were also evaluated using a 7-point scale (1 = “completely disagree,” 7 = “completely agree,” α = 0.90).

### 5.2 Result of Study 4

According to Hayes (2017), bootstrapping method was used to validate the chain mediation through Model 6 in Process. Game results as the independent variable, downward social comparison as the mediator variable 1, motivation to compete as the mediator variable 2, and willingness to download as the dependent variable are brought into the model, the bootstrap sample size is set to 5,000, and the confidence interval is set to 95%. As shown in Table 11, the main effect was supported, i.e., game results was able to significantly affect play willingness (B = 5.325, SE = 0.155, *p* < 0.001). Meanwhile, DSC was able to influence PW (B = 0.337, SE = 0.590, *p* < 0.001) and CM also influenced PW (B = 0.186, SE = 0.051, *p* < 0.001).

**Table 11:** Regression results of the chain mediating effects model (*n* = 326).

			*R* ^2^	*F*	*b*	SE	*t*	*p*	LLCI	ULCI
**Equation1**										
DSC	←	GR	0.113	41.402	2.632	0.232	11.329	0.000	2.175	3.089
**Equation2**										
CM	←	GR	0.226	47.214	1.782	0.227	7.844	0.000	1.335	2.229
	←	DSC			0.335	0.046	7.280	0.000	0.244	0.425
**Equation3**										
PW	←	GR	0.243	34.539	−0.837	0.131	−6.384	0.000	−1.554	−0.735
	←	DSC			0.377	0.590	6.434	0.000	0.262	0.492
	←	CM			0.186	0.051	3.610	0.000	0.085	0.287
**Equation4**										
PW	←	GR	0.238	101.110	5.325	0.155	34.302	0.000	−1.554	−0.735

Number of bootstrap samples for percentile bootstrap confidence intervals is 5,000. **p < 0.01 and ***p < 0.001.

**Figure 6:**
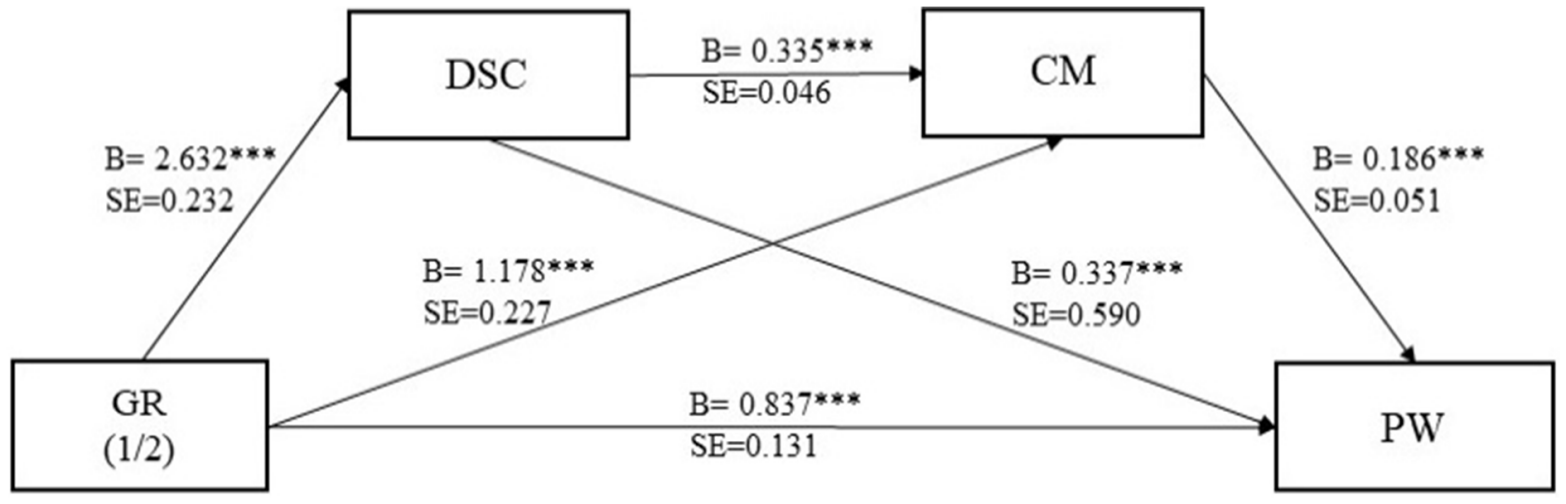
The maps of chain mediation model. **p < 0.01 and ***p < 0.001.

As shown in Table 12, the entire regression equation is highly significant. The total effect of GR on PW is −5.325 (SE = 0.155, *p* < 0.001). The direct effect coefficient is −1.145 (SE = 0.209, *p* < 0.001). The total indirect effect is −0.918, accounting for 80.17% (SE = 0.168, *p* < 0.001) of the total effect. Specifically, the indirect effects through DSC and CM paths contribute to 46.03%, with the DSC path accounting for 16.80%, and the CM path contributing 25.68%. Therefore, the research results confirm the significant chained mediation effects based on DSC and CM (see Figure 6).

**Table 12:** Results and comparison of chain mediating effect.

	Effect	BootSE	BootLLCI	BootULCI	Ratio of indirect to total effect (%)	Ratio of direct to total effect (%)
Total effect	−5.325	0.155	−1.554	−0.735		
Direct effect	−1.145	0.209	−1.554	−0.735		
Total indirect effect	−0.918	0.168	−1.300	−0.646	17.24	80.17
Ind1	−0.527	0.121	−0.807	−0.331	46.03	46.03
Ind2	−0.158	0.048	−0.431	−0.100	2.97	13.80
Ind3	−0.294	0.152	−0.626	−0.004	5.52	25.68

### 5.3 Discussion of Study 4

The results from Study 4 shed light on another facet of game advertising promotion. It involves showcasing players experiencing losses in the game to trigger downward social comparison among the audience, subsequently influencing their competitive motivation, and ultimately leading to an increased willingness to play. This mechanism stands apart from curiosity drive and is linked to the individual player’s actions. It’s crucial to note that witnessing others’ failures can also result in a partial loss of game-related information, such as knowledge of the correct progression path, weapon upgrades, level rewards, and game results screen. Consequently, this study hypothesized that within the mechanism of GR’s impact on PW, there might be an element of curiosity drive induced by this loss of information. Study 5 was developed by building on this analysis.

## 6 Study 5

Based on a previous research, a dual-path mechanism regarding the promotion of game advertising was delineated. One path involves the enhancement of players’ curiosity drive and, subsequently, an increase in play willingness through the perception of information gaps. The other path is through the influence of social comparison, which is triggered by game outcomes (win/lose) and impacts competitive motivation. Both paths independently contribute to an increase in play willingness. However, real-world game advertisements often employ a multifaceted promotional approach. Typically, game outcomes, such as winning or losing, often reflect disparities in information. The absence of a more comprehensive gaming experience and rewards at the culmination of failed game endeavors may stimulate participants’ curiosity drive. Hence, this study posits that game outcomes (win/lose) engender Information Missing Perception (IMP), subsequently instigating curiosity drive. Hence, the study proposed that game results (win/lose) lead to the Perception of Information Missing (PIM), consequently sparking curiosity. That is, the influence of game results (win/lose) on the willingness to play arises from the combined effects of downward social comparison and curiosity driven.

### 6.1 Research design

**Figure 7:**
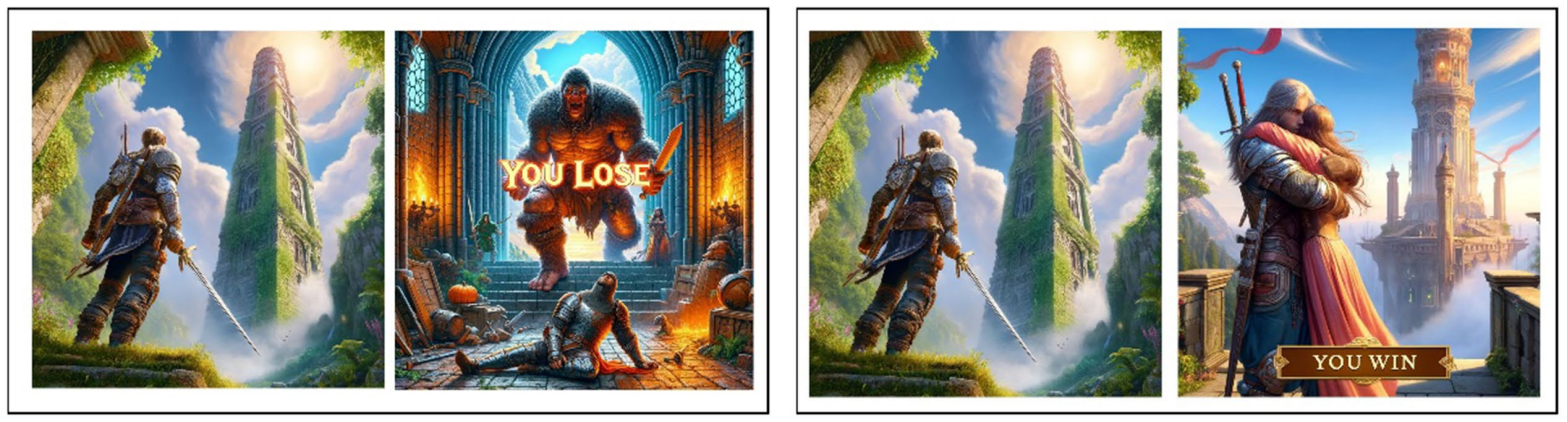
Left: the video of lose the game, right: the video of win the game. Note: Images generated by ChatGPT, version 4.0, response to “(Generate three game illustrations. 1. The warrior at the bottom of the tower. 2. The warrior is defeated by the monster in the tower, and YOU LOSE appears. 3. The warrior saves the princess, and YOU WIN appears)”, OpenAI (22 May 2024).

The tower climbing game from Study 3 was used as a sample in the design of two groups with game advertisements. In the experimental group advertisement, players intentionally challenged a boss beyond their level and failed, while in the control group advertisement, players challenged according to their level and ultimately succeeded (see Figure 7). Both groups of advertisements displayed clear settlement screens, with the only difference being the success or failure of the game outcome. The study expanded the age range of participants to assess the applicability of the advertisement’s effectiveness in different demographics. After removing two invalid samples, a total of 360 individuals completed the questionnaire (17–62 years old, M = 38.76, SD = 5.72; 172 females, 188 males).

After watching a 20-s video, participants were asked to complete a questionnaire that included measures of social comparison, competitive motivation, Information Missing Perception (IMP), and Curiosity Drive (CD). IMP was designed to assess whether or not the audience perceived the advertisement as lacking complete information, for example, “I feel like this advertisement is missing some content” and “I feel the video does not seem to capture all the aspects of the game.” CD was included to determine whether the perception of information missing could stimulate participants’ curiosity and interest (see Table 13).

**Table 13:** Measurement model and sources.

Construct	Items	Scale	References
Downward social comparison (DSC)	Compared to the players in the video, I think I can…	1–7 1 = “far worse” 7 = “much better”	Brown et al. (2007)
	Compared to the players in the video, I think my gaming skills are…		
	I’m more confident in my own gameplay compared to the players in the video.	1–7 1 = Very Disagree 7 = Very Agree	
	If I play this game, I’m sure I can do better.		
Competitive motivation (CM)	I want to do my best to outperform the players in the video.	1–7 1 = Very Disagree 7 = Very Agree	Cagiltay et al. (2015)
	I feel very confident to play better.		
	I want to prove that I’m a better player than the player in the video.		
Play willingness (PW)	I want to play this game	1–7 1 = Very Disagree 7 = Very Agree	Bijvank et al. (2012)
	I want to try out this game (Deleted)		
	I want to download this game		
	I want to control the in-game character myself		
Information missing perception (IMP)	I think the game video demo did not show the whole picture.	1–7 1 = Very Disagree 7 = Very Agree	NA
	I feel like this advertisement is missing some content.		
	I feel the video does not seem to capture all the aspects of the game.		
	I think the players in the video overlooked some elements.		
	I think the video is missing something important (Deleted).		
Curiosity driven (CD)	I’m curious about how it actually feels to play this game	1–7 1 = Very Disagree 7 = Very Agree	Renner (2006)
	I wonder what would happen if I played this game		
	I’m interested in the game’s level design		

### 6.2 Result of Study 5

Through AMOS structural equation modeling, this study examined the impact of the two dual pathways, IMP and DSC, on play willingness. Prior to hypothesis testing, the study established convergent validity (Table 14) and examined the interrelationships between factors (Table 15).

**Table 14:** Results of convergent validity (*n* = 326).

	Items	Unstd.	S.E.	*t*	*P*	Std.	SMC	CR	AVE
CD	CD1	1.000				0.832	0.692	0.876	0.703
	CD2	1.055	0.063	16.810	***	0.811	0.658		
	CD3	1.070	0.061	17.603	***	0.871	0.759		
CM	CM1	1.000				0.821	0.674	0.874	0.699
	CM2	1.160	0.068	16.984	***	0.849	0.721		
	CM3	1.026	0.061	16.850	***	0.837	0.701		
DSC	DSC1	1.000				0.770	0.593	0.863	0.616
	DSC2	1.008	0.059	17.053	***	0.892	0.796		
	DSC3	1.070	0.065	16.447	***	0.844	0.712		
	DSC4	0.862	0.076	11.308	***	0.602	0.362		
IMP	IMP1	1.000				0.835	0.697	0.861	0.612
	IMP2	0.879	0.053	16.657	***	0.805	0.648		
	IMP3	0.928	0.053	17.393	***	0.841	0.707		
	IMP4	0.838	0.068	12.277	***	0.628	0.394		
PW	PW1	1.000				0.851	0.724	0.898	0.746
	PW2	1.029	0.051	19.991	***	0.887	0.787		
	PW3	0.962	0.050	19.316	***	0.853	0.728		

***p < 0.001.

**Table 15:** Results of reliability and correlation matrix.

	Cronbach’α	AVE.	DSC	CM	PW	CD	IMP
DSC	0.852	0.616	**0.785**				
CM	0.875	0.699	0.642	**0.836**			
PW	0.845	0.746	0.515	0.541	**0.864**		
CD	0.873	0.703	0.167	0.197	0.427	**0.838**	
IMP	0.898	0.612	0.145	0.143	0.415	0.501	**0.782**

The bold diagonal is the square root value of average variance extracted (AVE).

As shown in Table 14, each tested item shows significant compliance with the requirements, and the standardized load coefficient values are all higher than 0.5 (Hair et al., 1987). The convergent validity of each construct was also supported (AVE ranged from 0.61 to 0.74 and CR ranged from 0.85 to 0.89).

In Table 15, the measured items within each construct demonstrated reliability (Cronbach’s α value >0.65). Additionally, the square roots of the Average Variance Extracted (AVE) values (found on the diagonal) for each latent variable exceeded the inter-construct correlations, confirming strong discriminant validity in the data.

The results of the structural equation model are shown in Table 16. It is evident that game results (win/lose) have an impact on Play Willingness (PW). Failed game demonstrations are more likely to increase an individual’s play willingness compared to successful game demonstrations (B = −0.521, SE = 0.117, *t* = −4.47, *p*<0.001). The mechanism of this advertising strategy is dual-pathed. On one hand, Game Results (GR) influence Information Missing Perception (IMP), meaning that failed game operations lead individuals to feel a sense of information deficiency (B = −0.601, SE = 0.112, *t* = −5.378, *p*<0.001). Then, IMP affects Curiosity Drive (CD), reinforcing the audience’s curiosity (B = 0.435, SE = 0.057, *t* = 7.667, *p*<0.001). Finally, CD further enhances play willingness (B = 0.251, SE = 0.069, *t* = 2.971, *p*<0.001).

**Table 16:** Results of influence paths.

Paths			Estimate	S.E.	*t*	*P*	*β*
IMP	←	GR	−0.601	0.112	−5.378	***	−0.297
DSC	←	GR	−0.923	0.122	−7.578	***	−0.404
CM	←	DSC	0.571	0.057	10.036	***	0.597
CD	←	IMP	0.435	0.057	7.667	***	0.470
CM	←	GR	−0.246	0.112	−2.203	0.028	−0.113
CD	←	GR	−0.201	0.099	−2.022	0.043	−0.107
PW	←	CD	0.251	0.069	3.623	***	0.199
PW	←	CM	0.302	0.070	4.325	***	0.280
PW	←	DSC	0.200	0.067	2.971	0.003	0.194
PW	←	GR	−0.521	0.117	−4.470	***	−0.221
PW	←	IMP	0.219	0.065	3.370	***	0.188

***p < 0.001.

On the other hand, game results (win/lose) trigger the audience’s Downward Social Comparison (DSC), with failed game operations being more effective at evoking DSC (B = −0.923, SE = 0.122, *t* = −7.578, *p*<0.001). DSC, in turn, influences Competitive Motivation (CM) (B = 0.571, SE = 0.057, *t* = 10.036, *p*<0.001). Finally, CM significantly impacts play willingness (B = 0.032, SE =0.07, *t* = 4.325, *p*<0.001).

Furthermore, as shown in Table 14, there are also indirect mediating effects in both of these pathways, and they are statistically significant. For example, GR directly affects CD (B = −0.201, SE = 0.099, *t* = −2.022, *p* = 0.043), while IMP can also directly influence PW (B = 0.219, SE = 0.065, *t* = 3.37, *p*<0.001). Similarly, GR directly impacts CM (B = −0.246, SE = 0.112, *t* = −2.203, *p* = 0.028), and DSC also has a direct effect on PW (B = 0.20, SE = 0.067, *t* = 2.971, *p*<0.003).

**Figure 8:**
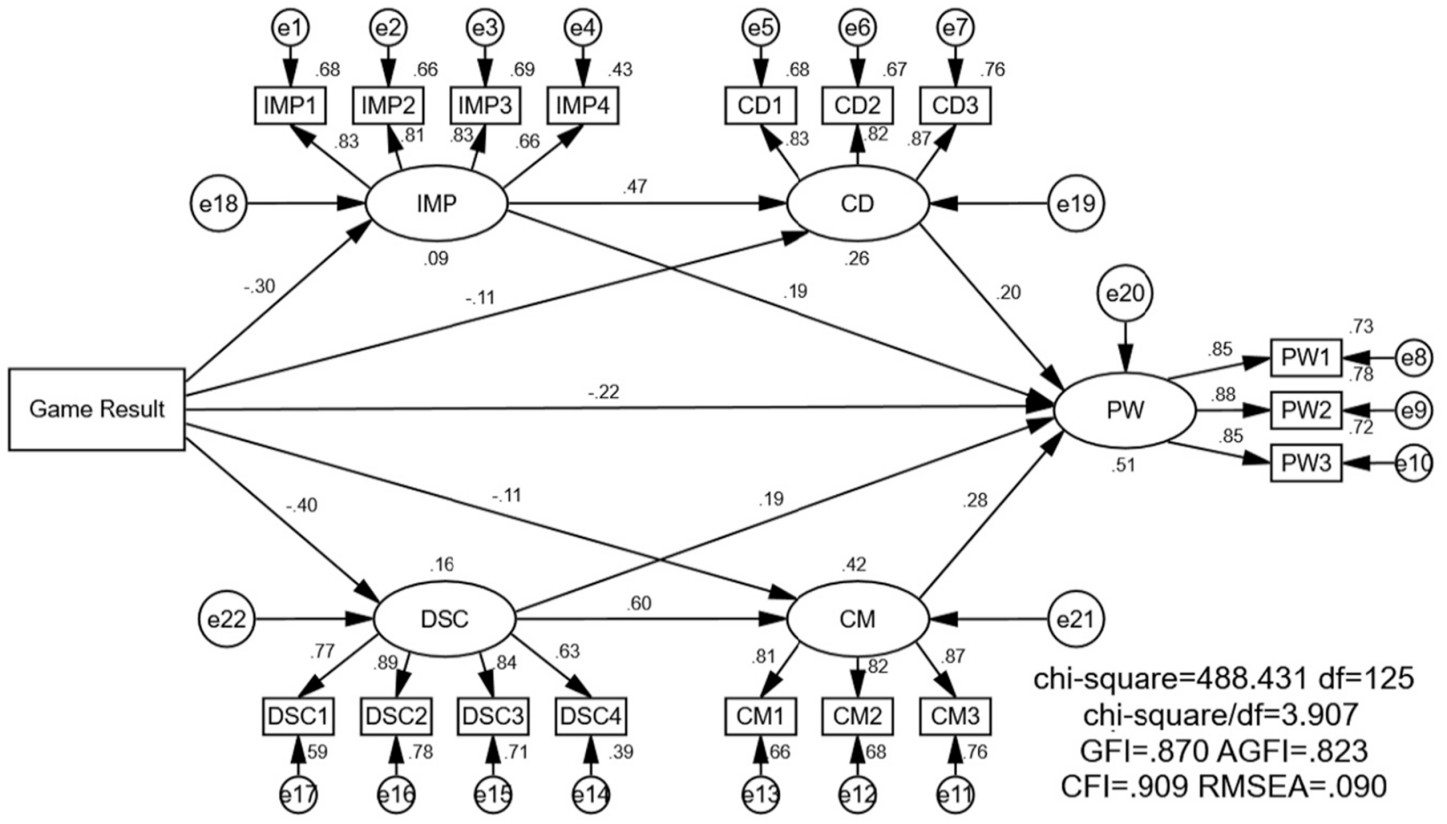
The structural model in AMOS.

As depicted in Figure 8, this study conducted model fitting and hypothesis testing using Amos 28.0. The model’s fit results and indices demonstrate a high level of adequacy. Specifically, *x*^2^/df = 3.907, GFI = 0.870, AFGI = 0.823, CFI = 0.909, RMSEA = 0.090. In accordance with the findings of Byrne (2001), all indices meet the reference standards, indicating a well-fitting model.

## 7 Limitations

Overall, this study conducted five experiments to investigate and reveal the cognitive mechanisms behind the strategies of information omission and showcasing failure in video game advertisements. However, the study has several limitations. First, the reliance on questionnaire-based assessments, while useful, is limited in capturing the full spectrum of conscious and unconscious cognitive evaluations (Suomala, 2020; Suomala and Kauttonen, 2023). Second, although questionnaires provide valuable insights into participants’ attitudes and self-reported behaviors, they may not fully capture the nuances of actual behavior in natural settings (Zadbood et al., 2021). Additionally, the methods employed may not accurately reflect the dynamic and multifaceted nature of cognitive processes as they occur in everyday life. Therefore, future research will incorporate qualitative analyses and utilize technologies such as EEG and eye-tracking to delve deeper into these processes and provide a more comprehensive understanding of cognitive dynamics.

## 8 Conclusion

This study intended to determine the effect of information missing in game videos on play willingness. In this regard, three experiments were conducted. Experiment 1 initially verified the main effect of game videos that did not show the ending, in which the game videos were more likely to stimulate play willingness than game videos that showed the ending. In Experiment 2, the psychological mechanism for the main effect was determined, namely the mediating role of curiosity drive. Specifically, game videos that did not show the outcome triggered additional curiosity in viewers, which in turn increased their willingness to play. Experiment 3 identified a moderating effect of NFCC, whereby an individual’s tolerance of message miss tolerance was the boundary condition for this effect. Specifically, individuals with high NFCC have the lower tolerance to videos with missing information, which strengthens the improvement of curiosity caused by missing information, thereby increasing the willingness to download. On the other hand, individuals with low NFCC have a higher tolerance to missing information, which weakens the improvement of curiosity caused by missing information, and thus reduces the willingness to download.

Another significant finding in this study is that witnessing others’ failures in game advertisements tends to increase audience gaming intentions more than observing successful game demonstrations. This is caused by a chain mediation of downward social comparison and competitive motivation. This phenomenon may also be related to a sense of “attainability of success.” When individuals see others fail in advertisement, they may perceive success in the game as more achievable and, consequently, feel more motivated to participate. In marketing and advertising strategies, capitalizing on this psychological effect can pique potential users’ interest and desire to engage. By showcasing players’ imperfect performances, advertisement may imply that the game is easy to grasp and can lead to quick accomplishments, attracting more players to try it. This strategy leverages consumers’ tendencies for downward social comparison and competition psychology, compelling them to prove their superior performance through gameplay. Furthermore, failed demonstrations reduce the exposure of game information and content, activating the curiosity drive, as seen in Study 5, further enhancing players’ willingness to play. This approach can also be effective for less competitive audiences.

### 8.1 Theoretical value

Numerous studies have confirmed that individuals can acquire knowledge and skills and increase their self-efficacy by observing the behaviour of others, especially their learning processes (Schunk and DiBenedetto, 2020). In particular, the integrity of external information is crucial, and the absence of content can divert attention and focus, which can have a negative effect on the development of individual interests (Golman and Loewenstein, 2015). However, games are recreational experiences where the focus is not on the acquisition of knowledge and skills, but on the psychological satisfaction achieved in the process. In order to pinpoint the cause of this phenomenon, Experiment 2 was designed and found that ‘curiosity’, as a mediating variable, drove viewers’ willingness to play. In light of this, the result not only supports the relationship that Berlyne (1962) proposed between curiosity and uncertainty (Berlyne, 1962; van Lieshout et al., 2021), but also establishes its applicability to game promotion.

Curiosity is also considered an ‘inconsistency or gap in knowledge’. An information gap arises when faced with a lack of information or uncertainty (Marvin and Shohamy, 2016). In general, most individuals cannot tolerate missing and ambiguous information, which can cause mild anxiety and boredom (Bar-Anan et al., 2009). Interestingly, some studies have found that seeking information to end ‘uncertainty’ can be pleasurable (van Lieshout et al., 2021). Therefore, this study argues that the lack of information in the game video creates information gap and the viewer increases play willingness in order to correct the lack and ambiguity. Hence the information gap leads to an increase in play willingness as an expression of the player’s pleasure in seeking to fill the information.

Moreover, the findings draw upon Bandura and Walters’ (1977) social learning theory and Schunk’s work in education psychology (Schunk, 2012; Schunk and DiBenedetto, 2020). In traditional educational settings, observing others’ successes has been associated with knowledge and skill acquisition. However, games are more about psychological satisfaction than skill acquisition, and this study demonstrates that witnessing failures in games can enhance curiosity and motivation. It extends the applicability of social learning theory to the domain of gaming promotion.

### 8.2 Practical value

Most previous research on gaming addiction focused on the individual’s interaction with the game, neglecting the mechanisms underlying the effect of watching others’ gaming behaviour (gaming videos) on the intention to play. In fact, with the widespread use of big data technologies, advertising has become more precise (Dianoux et al., 2014). The game advertising industry is also moving away from the traditional demonstration model and embracing the utilisation of streaming and short-form video platforms (Joa et al., 2018; Abbasi et al., 2021). This may have become a blasting fuse for gaming addiction in youth groups, especially college students. Therefore, identifying the mechanisms inherent in gaming video propaganda gives an early warning of gaming addiction in younger groups. It is more critical for individuals with high NFCC to avoid falling into the trap of curiosity-driven game propaganda than individuals with low NFCC.

Furthermore, the study provides insights into user behavior, offering potential strategies for improving advertising effectiveness. The findings suggest that highlighting others’ failures may attract users who are constantly exposed to idealized content on social media platforms, aligning with the concept of “Downward Social Comparison” (Wang et al., 2020). This knowledge can help advertisers tailor their content to specific audiences and maximize the impact of their advertising campaigns.

## Data availability statement

The datasets presented in this article are not readily available because the study involved a large sample size and significant investments of time and money, with concerns about potential misuse by commercial entities, but we are open to sharing with academic researchers upon request. Requests to access the datasets should be directed to DZ, daydzt@163.com.

## Ethics statement

Ethical review and approval were not required for the study on human participants in accordance with the local legislation and institutional requirements. Written informed consent from the participants was not required to participate in this study in accordance with the national legislation and the institutional requirements.

## Author contributions

DZ: Conceptualization, Data curation, Formal analysis, Funding acquisition, Investigation, Methodology, Project administration, Resources, Software, Supervision, Validation, Visualization, Writing – original draft, Writing – review & editing. XJ: Conceptualization, Data curation, Formal analysis, Funding acquisition, Investigation, Methodology, Project administration, Resources, Supervision, Validation, Visualization, Writing – original draft, Writing – review & editing. DJ: Data curation, Formal analysis, Methodology, Writing – original draft, Writing – review & editing. WH: Supervision, Validation, Visualization, Writing – original draft, Writing – review & editing.

## References

[ref1] Abbasi A. Z. Rehman U. Hussain A. Ting D. H. Islam J. U. (2021). The impact of advertising value of in-game pop-up ads in online gaming on gamers’ inspiration: an empirical investigation. Telematics Inform. 62:101630. doi: 10.1016/j.tele.2021.101630

[ref2] Adnan M. Abdullah J. M. Ibharim L. F. M. Hoe T. W. Janan D. Abdullah N.. (2019). Expanding opportunities for science, technology, engineering and mathematics subjects teaching and learning: connecting through comics. Malays. J. Med. Sci. 26, 127–133. doi: 10.21315/mjms2019.26.4.15, PMID: 31496902 PMC6719879

[ref3] Allen J. C. Jr. (2011). Sample size calculation for two independent groups: a useful rule of thumb. Proc. Singap. Healthc. 20, 138–140. doi: 10.1177/201010581102000213

[ref4] Apperley T. H. (2006). Genre and game studies: Toward a critical approach to video game genres. Simul. Gaming 37, 6–23. doi: 10.1177/1046878105282278

[ref5] Atkins B. (2003). More than a game: The computer game as fictional form. Waterloo Place, Oxford: Manchester University Press.

[ref6] Bandura A. Walters R. H. (1977). Social learning theory (vol. 1). Englewood Cliffs, NJ: Prentice Hall.

[ref500] Bandura A. (1982). Self-efficacy mechanism in human agency. Am Psychol. 37, 122. doi: 10.1037/0003-066X.37.2.122

[ref7] Bar-Anan Y. Wilson T. D. Gilbert D. T. (2009). The feeling of uncertainty intensifies affective reactions. Emotion 9:123. doi: 10.1037/a0014607 19186925

[ref8] Berlyne D. E. (1962). Uncertainty and epistemic curiosity. Br. J. Psychol. 53, 27–34. doi: 10.1111/j.2044-8295.1962.tb00811.x, PMID: 13867957

[ref501] Berlyne D. E. (1966). Curiosity and Exploration: animals spend much of their time seeking stimuli whose significance raises problems for psychology. Science, 153, 25–33. doi: 10.1126/science.153.3731.25 5328120

[ref9] Bijvank M. N. Konijn E. A. Bushman B. J. (2012). “We don’t need no education”: Video game preferences, video game motivations, and aggressiveness among adolescent boys of different educational ability levels. J. Adolesc. 35, 153–162. doi: 10.1016/j.adolescence.2011.04.001 21529925

[ref10] Bolin J. H. (2014). Reviewed work: Introduction to mediation, moderation, and conditional process analysis: a regression-based approach Andrew F. Hayes. J. Educ. Meas. 51, 335–337. doi: 10.1111/jedm.12050

[ref11] Brown D. J. Ferris D. L. Heller D. Keeping L. M. (2007). Antecedents and consequences of the frequency of upward and downward social comparisons at work. Organ. Behav. Hum. Decis. Process. 102, 59–75. doi: 10.1016/j.obhdp.2006.10.003

[ref12] Byrne B. M. (2001). Structural equation modeling with AMOS, EQS, and LISREL: comparative approaches to testing for the factorial validity of a measuring instrument. Int. J. Test. 1, 55–86. doi: 10.1207/S15327574IJT0101_4

[ref13] Cagiltay N. E. Ozcelik E. Ozcelik N. S. (2015). The effect of competition on learning in games. Comput. Educ. 87, 35–41. doi: 10.1016/j.compedu.2015.04.001

[ref14] Chen C. W. (2018). An innovative board game design based on cross-cultural communication. Des. Culture 10, 209–217. doi: 10.1080/17547075.2018.1467723

[ref15] Chiu C. Y. Morris M. W. Hong Y. Y. Menon T. (2000). Motivated cultural cognition: the impact ofimplicit cultural theories on dispositional attribution varies as a function of need for cognitive closure. J. Pers. Soc. Psychol. 78, 247–259. doi: 10.1037/0022-3514.78.2.247, PMID: 10707332

[ref16] Collins R. P. Litman J. A. Spielberger C. D. (2004). The measurement of perceptual curiosity. Pers. Indiv. Differ. 36, 1127–1141. doi: 10.1016/S0191-8869(03)00205-8

[ref17] Connolly T. M. Boyle E. A. MacArthur E. Hainey T. Boyle J. M. (2012). A systematic literature review of empirical evidence on computer games and serious games. Comput. Educ. 59, 661–686. doi: 10.1016/j.compedu.2012.03.004

[ref18] Daume J. Hüttl-Maack V. (2020). Curiosity-inducing advertising: how positive emotions and expectations drive the effect of curiosity on consumer evaluations of products. Int. J. Advert. 39, 307–328. doi: 10.1080/02650487.2019.1633163

[ref19] Dianoux C. Linhart Z. Vnoucková L. (2014). Attitude toward advertising in general and attitude toward a specific type of advertising-a first empirical approach. J. Compet. 6, 87–103. doi: 10.7441/joc.2014.01.06

[ref20] Diel K. Hofmann W. (2019). Inspired to perspire: the interplay of social comparison direction and standard extremity in the context of challenging exercising goals. Soc. Cogn. 37, 247–265. doi: 10.1521/soco.2019.37.3.247

[ref502] Diel K. Grelle S. Hofmann W. (2021). A motivational framework of social comparison. J. Pers. Soc. Psychol, 120, 1415. doi: 10.1037/pspa0000204 33507785

[ref21] Eccles J. S. Wigfield A. (2020). From expectancy-value theory to situated expectancy-value theory: a developmental, social cognitive, and sociocultural perspective on motivation. Contemp. Educ. Psychol. 61:101859. doi: 10.1016/j.cedpsych.2020.101859

[ref22] Faul F. Erdfelder E. Lang A. G. Buchner A. (2007). G* Power 3: A flexible statistical power analysis program for the social, behavioral, and biomedical sciences. Behav. Res. Methods 39, 175–191. doi: 10.3758/BF03193146 17695343

[ref23] Festinger L. (1954). A theory of social comparison processes. Hum. Relat. 7, 117–140. doi: 10.1177/001872675400700202

[ref24] Galdieri R. Haggis-Burridge M. Buijtenweg T. Carrozzino M. (2020). “Exploring players’ curiosity-driven behaviour in unknown videogame environments” in International conference on augmented reality, virtual reality and computer graphics. eds. De Paolis L. Bourdot P. (Cham: Springer), 177–185.

[ref25] Gibbons F. X. Lane D. J. Gerrard M. Reis-Bergan M. Lautrup C. L. Pexa N. A.. (2002). Comparison-level preferences after performance: Is downward comparison theory still useful? J. Pers. Soc. Psychol. 83:865. doi: 10.1037/0022-3514.83.4.865 12374441

[ref26] Golman R. Loewenstein G. (2015). Curiosity, information gaps, and the utility of knowledge. Information gaps, and the utility of knowledge (April 16, 2015), 96–135.

[ref27] Gong X. Cheung C. M. Zhang K. Z. Chen C. Lee M. K. (2021). A dual-identity perspective of obsessive online social gaming. J. Assoc. Inf. Syst. 22, 1245–1284. doi: 10.17705/1jais.00693

[ref28] Hair J. F. Anderson R. E. Tatham R. L. Black W. C. (1987). Multivariate data analysis with readings. New York: MacMillan.

[ref29] Hauke N. Abele A. E. (2020). Two faces of the self: actor-self perspective and observer-self perspective are differentially related to agency versus communion. Self Identity 19, 346–368. doi: 10.1080/15298868.2019.1584582

[ref30] Hayes A. F. (2017). Introduction to mediation, moderation, and conditional process analysis. Philadelphia: A regression-based approach: Guilford Publications.

[ref31] Hill K. M. Fombelle P. W. Sirianni N. J. (2016). Shopping under the influence of curiosity: how retailers use mystery to drive purchase motivation. J. Bus. Res. 69, 1028–1034. doi: 10.1016/j.jbusres.2015.08.015

[ref32] Hornikx J. Mulder E. (2015). The curiosity-evoking capacity of foreign languages in advertising. Dutch J. Appl. Linguist. 4, 59–66. doi: 10.1075/dujal.4.1.05hor

[ref33] Joa C. Y. Kim K. Ha L. (2018). What makes people watch online in-stream video advertisements? J. Interact. Advert. 18, 1–14. doi: 10.1080/15252019.2018.1437853

[ref34] Kang M. J. Hsu M. Krajbich I. M. Loewenstein G. McClure S. M. Wang J. T. Y.. (2009). The wick in the candle of learning: Epistemic curiosity activates reward circuitry and enhances memory. Psychol. Sci. 20, 963–973. doi: 10.1111/j.1467-9280.2009.02402.x 19619181

[ref35] Kashdan T. B. Silvia P. J. (2009). “Curiosity and interest: the benefits of thriving on novelty and challenge” in Oxford handbook of positive psychology. eds. Lopez S. J. Snyder C. R.. 2nd ed (Oxford: Oxford University Press), 367–374.

[ref36] Kemmelmeier M. (2015). The closed-mindedness that wasn’t: Need for structure and expectancy-inconsistent information. Front. Psychol. 6:135179. doi: 10.3389/fpsyg.2015.00896 PMC4488610 26191017

[ref37] Kim D. Y. Kim H. Y. (2021). Influencer advertising on social media: the multiple inference model on influencer-product congruence and sponsorship disclosure. J. Bus. Res. 130, 405–415. doi: 10.1016/j.jbusres.2020.02.020

[ref38] Kruglanski A. W. (2013). Lay epistemics and human knowledge: Cognitive and motivational bases. New York, NY: Springer Science & Business Media.

[ref39] Kruglanski A. W. Webster D. M. Klem A. (1993). Motivated resistance and openness to persuasion in the presence or absence of prior information. J. Pers. Soc. Psychol. 65:861. doi: 10.1037/0022-3514.65.5.861 8246114

[ref40] Lee H. S. Griffith D. A. (2019). Social comparison in retailer–supplier relationships: referent discrepancy effects. J. Mark. 83, 120–137. doi: 10.1177/0022242918823542

[ref41] Lee C. H. Kim H. R. (2022). Positive and negative switching barriers: promoting hotel customer citizenship behaviour through brand attachment. Int. J. Contemp. Hosp. Manag. 34, 4288–4311. doi: 10.1108/IJCHM-10-2021-1280

[ref42] Litman J. (2005). Curiosity and the pleasures of learning: Wanting and liking new information. Cogn. Emot. 19, 793–814.

[ref43] Litman J. A. Spielberger C. D. (2003). Measuring epistemic curiosity and its diversive and specific components. J. Pers. Assess. 80, 75–86. doi: 10.1207/S15327752JPA8001_16, PMID: 12584070

[ref44] Marvin C. B. Shohamy D. (2016). Curiosity and reward: valence predicts choice and information prediction errors enhance learning. J. Exp. Psychol. Gen. 145, 266–272. doi: 10.1037/xge0000140, PMID: 26783880

[ref45] McStay A. (2017). Digital advertising. New York, NY: Bloomsbury Publishing.

[ref46] Moisieiev D. Dimitriu R. Jain S. P. (2020). So happy for your loss: consumer schadenfreude increases choice satisfaction. Psychol. Mark. 37, 1525–1538. doi: 10.1002/mar.21399

[ref47] Nagle A. Wolf P. Riener R. Novak D. (2014). The use of player-centered positive reinforcement to schedule in-game rewards inreases enjoyment and performance in a serious game. Int. J. Serious Games 1, 35–47. doi: 10.17083/ijsg.v1i4.47

[ref48] Oliver M. B. Bowman N. D. Woolley J. K. Rogers R. Sherrick B. I. Chung M. Y. (2016). Video games as meaningful entertainment experiences. Psychol. Pop. Media Cult. 5:390. doi: 10.1037/ppm0000066

[ref49] Pahlevan Sharif S. She L. Yeoh K. K. Naghavi N. (2022). Heavy social networking and online compulsive buying: the mediating role of financial social comparison and materialism. J. Mark. Theory Pract. 30, 213–225. doi: 10.1080/10696679.2021.1909425

[ref50] Paiva J. C. Leal J. P. Queirós R. (2020). Authoring game-based programming challenges to improve students’ motivation. In The Challenges of the Digital Transformation in Education: Proceedings of the 21st International Conference on Interactive Collaborative Learning (ICL2018)-Volume 1 (pp. 602-613). Springer International Publishing.

[ref51] Pierro A. Kruglanski A. W. (2008). “Seizing and freezing” on a significant-person schema: need for closure and the transference effect in social judgment. Personal. Soc. Psychol. Bull. 34, 1492–1503. doi: 10.1177/0146167208322865, PMID: 18948431

[ref52] Pierro A. Mannetti L. Converso D. Garsia V. Miglietta A. Ravenna M.. (1995). Caratteristiche strutturali della versione italiana di Bisogno di Chiusura Cognitiva (di Webster & Kruglanski) [structural characteristics of the Italian version of the need for cognitive closure scale (of Webster & Kruglanski)]. TPM: testing. Psicometria, Meto-dologia 2, 125–142.

[ref53] Przybylski A. K. Rigby C. S. Ryan R. M. (2010). A motivational model of video game engagement. Rev. Gen. Psychol. 14, 154–166. doi: 10.1037/a0019440

[ref54] Renner B. (2006). Curiosity about people: The development of a social curiosity measure in adults. J. Pers. Assess. 87, 305–316. doi: 10.1207/s15327752jpa8703_11 17134338

[ref55] Roets A. Kruglanski A. W. Kossowska M. Pierro A. Hong Y. Y. (2015). “The motivated gatekeeper of our minds: New directions in need for closure theory and research” in Advances in experimental social psychology (vol. 52). Eds. Zanna M. P. Olson J. M. (London: Academic Press), 221–283.

[ref56] Salanova M. Martinez I. M. Llorens Gumbau S. (2012). Success breeds success, especially when self-efficacy is related with a causality internal attribution. Stud. Psychol. 33, 151–165. doi: 10.1174/021093912800676420

[ref57] Sankey K. S. Machin M. A. (2014). Employee participation in non‐mandatory professional development–the role of core proactive motivation processes. Int. J. Train. Dev. 18, 241–255. doi: 10.1111/ijtd.12036

[ref58] Schunk D. H. (2012). Learning theories an educational perspective. Hoboken, United states: Pearson Education, Inc.

[ref59] Schunk D. H. DiBenedetto M. K. (2020). Motivation and social cognitive theory. Contemp. Educ. Psychol. 60:101832. doi: 10.1016/j.cedpsych.2019.101832

[ref60] Seo Y. N. Kim M. Lee D. Jung Y. (2018). Attention to eSports advertisement: effects of ad animation and in-game dynamics on viewers’ visual attention. Behav. Inform. Technol. 37, 1194–1202. doi: 10.1080/0144929X.2018.1488993

[ref61] Steffen C. Mau G. Schramm-Klein H. (2013). Who is the loser when I lose the game? Does losing an advergame have a negative impact on the perception of the brand? J. Advert. 42, 183–195. doi: 10.1080/00913367.2013.774598

[ref62] Suomala J. (2020). The consumer contextual decision-making model. Front. Psychol. 11:570430. doi: 10.3389/fpsyg.2020.570430, PMID: 33117237 PMC7559398

[ref63] Suomala J. Kauttonen J. (2023). Computational meaningfulness as the source of beneficial cognitive biases. Front. Psychol. 14:1189704. doi: 10.3389/fpsyg.2023.1189704 37205079 PMC10187636

[ref64] Tan W. H. Johnston-Wilder S. Neill S. (2010). Exploring the educational potential of game-based learning through the eyes of game industry practitioners. Int. J. Technol. Knowl. Soc. 6, 41–54. doi: 10.18848/1832-3669/CGP/v06i01/56058

[ref65] Tjosvold D. Johnson D. W. Johnson R. T. Sun H. (2006). Competitive motives and strategies: Understanding constructive competition. Group Dyn. Theory Res. Pract. 10:87. doi: 10.1037/1089-2699.10.2.87

[ref66] To A. Ali S. Kaufman G. Hammer J. (2016). Integrating curiosity and uncertainty in game design. Digra/fdg.

[ref503] Toteva I. T. Lutz R. J. Shaw E. H. (2021). The curious case of productivity orientation: The influence of advertising stimuli on affect and preference for subscription boxes. J. Retail. Consum. Serv, 63, 102677. doi: 10.1016/j.jretconser.2021.102677

[ref67] van Lieshout L. L. de Lange F. P. Cools R. (2021). Uncertainty increases curiosity, but decreases happiness. Sci. Rep. 11, 1–10. doi: 10.1038/s41598-021-93464-6 34234250 PMC8263743

[ref68] Wang W. Wang M. Hu Q. Wang P. Lei L. Jiang S. (2020). Upward social comparison on mobile social media and depression: The mediating role of envy and the moderating role of marital quality. J. Affect. Disord. 270, 143–149. doi: 10.1016/j.jad.2020.03.173 32339106

[ref69] Weary G. Elbin S. Hill M. G. (1987). Attributional and social comparison processes in depression. J. Pers. Soc. Psychol. 52, 605–610. doi: 10.1037/0022-3514.52.3.605 3572728

[ref504] Webster D. M. (1993). Motivated augmentation and reduction of the overattribution bias. J. Pers. Soc. Psychol. 65:261. doi: 10.1037/0022-3514.65.2.261, PMID: 8366420

[ref70] Webster D. M. Kruglanski A. W. (1994). Individual differences in need for cognitive closure. J. Pers. Soc. Psychol. 67, 1049–1062. doi: 10.1037/0022-3514.67.6.1049 7815301

[ref71] Wong Y. S. Yatim M. H. M. Tan W. H. (2014). Use computer game to learn object-oriented programming in computer science courses. In 2014 IEEE global engineering education conference (EDUCON) (pp. 9–16). IEEE.

[ref72] Wouters P. Van Oostendorp H. Boonekamp R. Van der Spek E. (2011). The role of game discourse analysis and curiosity in creating engaging and effective serious games by implementing a back story and foreshadowing. Interact. Comput. 23, 329–336. doi: 10.1016/j.intcom.2011.05.001

[ref73] Xi N. Hamari J. (2019). Does gamification satisfy needs? A study on the relationship between gamification features and intrinsic need satisfaction. Int. J. Inf. Manage. 46, 210–221. doi: 10.1016/j.ijinfomgt.2018.12.002

[ref505] Yucel-Aybat O. Kramer T. (2017). Comparative advertisements and schadenfreude: when and why others’ unfortunate choices make us happy. Mark. Lett. 28, 579–589. doi: 10.1007/s11002-017-9431-8

[ref506] Yucel-Aybat O. Kramer T. (2018). The impact of competitiveness on consumer responses to comparative advertisements. J. Advert. 47, 198–212. doi: 10.1080/00913367.2018.1430624

[ref74] Zadbood A. Nastase S. A. Chen J. Norman K. A. Hasson U. (2021). Here’s the twist: How the brain updates the representations of naturalistic events as our understanding of the past changes. Bio Rxiv, 2021–09. doi: 10.7554/eLife.79045 PMC9842385 36519530

